# Photoprotective Potential of the Natural Artocarpin against In Vitro UVB-Induced Apoptosis

**DOI:** 10.1155/2020/1042451

**Published:** 2020-09-19

**Authors:** Kunlathida Luangpraditkun, Pensri Charoensit, François Grandmottet, Céline Viennet, Jarupa Viyoch

**Affiliations:** ^1^Department of Pharmaceutical Technology, Faculty of Pharmaceutical Sciences and Center of Excellence for Innovation in Chemistry, Naresuan University, Phitsanulok 65000, Thailand; ^2^Department of Biochemistry, Faculty of Medical Science, Naresuan University, Phitsanulok 65000, Thailand; ^3^UMR 1098 RIGHT INSERM EFS BFC, University of Bourgogne Franche-Comté, Besançon 25000, France

## Abstract

Apoptosis, a well-known pattern of programmed cell death, occurs in multicellular organisms not only for controlling tissue homeostasis but also for getting rid of severely damaged cells in order to protect the redundant growth of abnormal cells undergoing cancerous cells. The epidermis of the human skin, composed largely of keratinocytes (KCs), is renewed continuously. Therefore, KCs apoptosis plays a critical role in the maintenance of epidermis structure and function. However, regulated cell death can be disturbed by environmental factors especially ultraviolet radiation (UV) B, leading to the formation of sunburn cells (KCs undergoing UVB-induced apoptosis) and impairing the skin integrity. In the present study, we firstly reported the potential of the natural artocarpin (NAR) to regulate UVB-induced human KCs apoptosis. The NAR showed antilipid peroxidation with an IC_50_ value of 18.2 ± 1.6 *μ*g/mL, according to TBARS assay while the IC_50_ value of trolox, a well-known antioxidant, was 7.3 ± 0.8 *μ*g/mL. For cell-based studies, KCs were pretreated with 3.1 *μ*g/mL of the NAR for 24 hr and then exposed to UVB at 55 mJ/cm^2^. Our data indicated that the NAR pretreatment reduces UVB-induced oxidative stress by scavenging free radicals and nitric oxide and therefore prevents reactive oxygen species (ROS) and reactive nitrogen species- (RNS-) mediated apoptosis. The NAR pretreatment has been shown also to reduce the UVB-induced cyclobutane pyrimidine dimer (CPD) lesions by absorbing UVB radiation and regulating the cell cycle phase. Additionally, the NAR pretreatment was found to modulate the expression of cleaved caspases-3 and 8 that trigger different signalling cascades leading to apoptosis. Thus, these results provide a basis for the investigation of the photoprotective effect of the NAR isolated from *A. altilis* heartwood and suggest that it can be potentially used as an agent against UVB-induced skin damages.

## 1. Introduction

Apoptosis or a pattern of programmed cell death occurs in multicellular organisms. It is important not only for controlling tissue homeostasis but also for getting rid of severely damaged cells to mediate protection against tumor and cancer developments [1, 2]. For the skin, proliferation, differentiation, and cell death of keratinocytes (KCs) must be controlled for supporting tissue function and preventing redundant growth. The regulation of cell death is very essential for maintaining cutaneous homeostasis.

The skin is the main target of ultraviolet (UV) radiation and UVB is considered the most damaging and genotoxic component of sunlight [3]. UVB induces various cell modifications including mutations in DNA, cell cycle arrest, apoptotic responses through various signalling pathways, and formation of reactive oxygen species (ROS). These effects cause multiple physiological events, such as erythema and inflammation [4, 5]. Particularly, one of the main biological features of acute and chronic exposure of skin cells to UVB is the induction of apoptotic KCs [6, 7]. DNA damage, cell surface death receptor activation, and formation of free radicals have been shown to be involved [8–10]. The direct effect of UVB is the absorption of photons by DNA generating DNA lesions which include cyclobutane pyrimidine dimers (CPD) and pyrimidine -pyrimidone (6-4) photoproducts (6-4 PPs) [2, 4]. In cellular responses, KCs arrest cell cycle in the G1 phase in order to repair damaged DNA before its replication in the S phase. However, if the repair of DNA lesions is not efficient, caspases are activated and generate a cascade of signalling events leading to apoptosis [8, 11]. The indirect effect of UVB is the excessive production of free radicals, both reactive oxygen species (ROS) such as superoxide radicals and reactive nitrogen species (RNS) such as nitric oxide. Free radicals disrupt redox homeostasis in the skin and trigger severe oxidative stress. They cause damage to proteins, nucleic acids, lipids, membranes, and mitochondria and activate the death receptor pathway of apoptosis [10, 12, 13]. All these events, linked to the disruption of the DNA repair mechanism and the antioxidant defense, can lead to the activation of cell death processes such as apoptosis.

Most of the apoptotic pathways bring about cysteine-dependent aspartate-specific protease (caspases) activation, especially those in response to UVB [14]. Two main apoptotic pathways were reported including the intrinsic pathway which involves upstream initiators such as caspase-9 and the extrinsic pathway which involves upstream initiators such as caspase-8. Once upstream initiator caspases are st**i**mulated, downstream effectors such as caspase-3 are activated and regulate apoptosis [12, 15]. In addition, UVB can activate the transcription and release of proinflammatory cytokines including tumor necrosis factor- (TNF-) *α* through the NF*κ*B signalling pathway [16]. Especially, TNFTNF-*α* can further upregulate other cytokines affecting inflammatory skin through the mitogen-activated protein kinase (MAPK) signalling pathway including the p38 MAPK and JNK signalling pathway [17].

To prevent UVB-induced skin damages, it is useful to develop biologically active drugs with antioxidant, anti-inflammatory, and antiapoptotic properties. Many studies have shown great potential of natural substances on different in vitro cell lines and in vivo experimental animal models. *Artocarpus altilis* heartwood extract has been reported to possess many biological activities such as antioxidant [18–23], anti-inflammatory [6, 18, 22], antityrosinase [19], and antimelanogenic activities [19, 21, 23]. Furthermore, it has been shown that artocarpin attenuates UVB-induced photodamage in hairless mice through antioxidant and anti-inflammatory effects [22]. The anti-inflammatory activity is associated with its ability to suppress the production of TNF-*α* in UVB-irradiated human KCs [18].

The present work was aimed at investigating the ability of the artocarpin isolated from breadfruit heartwood (the natural artocarpin (NAR)) to attenuate UVB-induced human KC apoptosis. We studied the effect of NAR on UVB-induced human KCs in vitro through apoptotic markers including the formation of ROS and RNS, the occurrence of DNA lesions, and the expression of caspase proteins.

## 2. Materials and Methods

### 2.1. Isolation of the Natural Artocarpin

Fresh heartwood of a breadfruit tree (*Artocarpus altilis*) was collected during October at Phitsanulok Province, Thailand. The heartwood was chopped into small pieces and then dried at 45°C for 3 days. The dried pieces were macerated with diethyl ether at room temperature for 2 cycles (2 days/cycle) as explained previously [18]. The ether extract was concentrated by using rotary evaporation and then flowed through a column chromatography packed with silica gel (No. 60, Merck Millipore, Darmstadt, Germany). Hexane : ethyl acetate (gradient) was used as the eluting solvent. The presence of flavonoid compounds was identified in the fractions by using a thin-layer chromatography (Merck Millipore, Darmstadt, Germany). The organic solvents were removed from the collected fractions by using rotary evaporation, and the obtained sample was called the natural artocarpin (NAR). All solvents used were of analytical grade and purchased from Labscan Asia Co., Ltd., Bangkok, Thailand.

### 2.2. NAR Qualification

The chemical structure of the obtained NAR was determined by using Nuclear Magnetic Resonance (NMR) spectroscopy (Bruker Avance 400, Bruker Corporation, Massachusetts, USA). Moreover, the content of artocarpin in the NAR was examined by using isocratic high-performance liquid chromatography (HPLC model LC 20AP, Shimadzu Co., Ltd., Kyoto, Japan) as explained previously [18]. A column packing with C18 (Phenomenex Gemini column) and mobile phase (MP) consisting of methanol (HPLC grade, Labscan Asia Co., Ltd.) and water (80 : 20) was the selected system. The amount of artocarpin in the NAR was calculated using a calibration curve generated from the standard artocarpin (BioBioPha Co., Ltd., Yunnan, China).

### 2.3. Antilipid Peroxidation Activity of the NAR

To determine the antilipid peroxidation activity of the NAR, thiobarbituric acid- (TBA-) reactive substance (TBARS) assay was used [24]. Briefly, mouse brain homogenate (substrate) (Committee on animal care and use for scientific work of Naresuan University, approval no. NU-AEE620808) was incubated with the NAR or trolox (Sigma-Aldrich, Missouri, USA) at various concentrations. FeSO_4_ (analytical reagent (AR) grade, Fisher Scientific U.K. Ltd. Loughborough, United Kingdom) and ascorbic acid (AR grade, Chem-Supply Pty Ltd, Gillman, Australia) were then added. The resultant mixtures were reacted at 37°C for 45 min. The reaction was further propagated by adding SDS (Calbiochem®, EMD Millipore Corporation, Darmstadt, Germany), acetic acid (AR grade, RCI Labscan Ltd, Thailand), and TBA (AR grade, Sigma-Aldrich, Darmstadt, Germany), and the mixture was incubated at 90°C for 1 hr. After that, samples were centrifuged, and supernatants were collected to measure the amount of malondialdehyde- (MDA-) BARS complex by using a microplate reader (BioStack Ready, BioTek Instruments, Vermont, USA) at 532 nm. The antilipid peroxidation activity of the NAR and trolox was determined from the following equation:

(1) $$\begin{array}{c} \%inhibition\;\text{of }lipid\;peroxidation=\left[\left.\left(100-As\right)/Ac\right)\right]\times 100. \end{array}$$

where *A*_c_ is an absorbance of control and *A*_s_ is an absorbance of the sample

Three independent experiments were performed in this study. Each experiment was repeated in triplicate (*n* = 3).

### 2.4. Isolation and Cultivation of Human Keratinocytes (KCs)

KCs were isolated from excess eyelid skin removed from women aged 50-60 years old who underwent elective cosmetic surgery. We expected that if the NAR possesses the ability to be a naturally photoprotective agent on the photodamaged and weak cells isolated from the elder donors, we can develop antiphotoaging skin care products for old adults as major consumers. In this study, the protocol was approved by the Institutional Review Board of Naresuan University (approval code: COA no. 356/2016). The skin tissues were cleaned with phosphate-buffered saline (PBS) containing antibiotics (100 units/mL penicillin, 100 mg/mL streptomycin, and 1 mg/mL amphotericin B (Gibco™, Invitrogen, New York, USA) and incubated in 5% dispase solution (Gibco™, Godo Shusei Co., Ltd., Tokyo, Japan) overnight at 4°C. KCs were isolated from the epidermis layer and cultured using previous reports [25, 26] with modifications. Briefly, small pieces of the epidermal layer were incubated in 0.25% trypsin–EDTA solution (Gibco®, Ontario, Canada) for 10 min at 37°C. The collected cells were seeded at 2 × 10^4^ cells/cm^2^ in 75 cm^2^ flasks containing keratinocyte-serum-free medium (K-SFM, Gibco, New York, USA) supplemented with epidermal growth factor 1-53 (EGF1-53), bovine pituitary extract (BPE), and antibiotics at 37°C in a humid atmosphere containing 5% CO_2_. The medium was changed every 2-3 days. KCs were subcultured at 80% of cell confluence, and passage numbers 1 to 4 were used in this study.

### 2.5. UVB Exposure

KCs were seeded in 96-well plates (1 × 10^4^ cells/well, density/cm^2^ = 3.1 × 10^4^) or 24-well plates (6 × 10^4^ cells/well, density/cm^2^ = 3.1 × 10^4^) and grown in medium with supplements and antibiotics in a CO_2_ incubator for 24 hr. Next, KCs were cultured in supplement-free medium for another 24 hr. Prior to UVB exposure, cells were washed with PBS. Cells were exposed to various intensities of UVB (27.5, 55, and 110 mJ/cm^2^) under a thin layer of PBS. The UVB source emitted light at 275–305 nm (Toshiba FL8BLB, Japan) and was positioned 12 cm above the cell culture plate. The UVB intensity was determined using a UVB meter (Solarmeter, Solartech, Pennsylvania, USA). After exposure, cells were further incubated in supplement-free medium for 10 or 24 hr, and cell viability and apoptosis were determined.

### 2.6. Cell Viability Assay

Cell viability of KCs after UVB exposure at various intensities was determined using XTT assay (Roche Diagnostics Corporation, Indianapolis, USA) [18]. Briefly, after UVB exposure for 10 or 24 hr, 50 *μ*L of serum-free medium and 50 *μ*L of XTT solution were added and incubated for 4 hr. The cell viability was quantified by measuring the absorbance at 490 nm using a microplate reader (BioStack Ready, BioTek instruments, Vermont, USA). The absorbance of the non-UVB-exposed cells was adjusted to 100% viability. Cell morphology was observed by using an inverted microscope (AE2000TRI, Motics, China). Three independent experiments were performed in this study. Each experiment was repeated in triplicate (*n* = 3).

### 2.7. Cell Apoptosis Analysis

Annexin V/7-AAD staining (Nexin reagent kit, Merck, Millipore, Darmstadt, Germany) was used to detect apoptotic cells after UVB exposure. After 10 or 24 hr of irradiation, the UVB-irradiated cells were stained with Nexin reagent kit, according to the manufacturer's instructions. Briefly, cells were resuspended with 1% FBS in 1X assay buffer; then, 100 *μ*L of cell suspension was mixed with 100 *μ*L of Muse™ Annexin V and dead cell reagent and incubated for 20 min at room temperature. The percentages of viable (VA), total apoptotic (TA), and necrotic cells (NC) were measured using flow cytometry (Guava® easyCyte™, Merck Millipore, Massachusetts, USA). Three independent experiments were performed in this study. Each experiment was repeated in triplicate (*n* = 3).

### 2.8. Photoprotective Effect of the NAR on UVB-Induced KCs Apoptosis

#### 2.8.1. Pretreatment of KCs with the NAR

For analysis of apoptotic cells, ROS, RNS, and cell cycle distribution, KCs were seeded in 24-well plates (6 × 10^4^ cells/well, cell density/cm^2^ = 3.1 × 10^4^). For the study of apoptosis-related protein expression and cyclobutane pyrimidine dimers (CPDs) and 6-4 photoproducts (PPs) induction, cells were seeded in 75 cm^2^ culture flask (2.3 × 10^6^ cells/flask, cell density/cm^2^ = 3.1 × 10^4^). KCs were cultured in medium with supplements in a CO_2_ incubator for 24 hr. KCs were then pretreated with supplement-free medium containing the NAR (3.1 *μ*g/mL, solubilized in 0.1% DMSO) in the CO_2_ incubator for 24 hr. After that, cells were washed with PBS, exposed to UVB at the desired intensity (55 mJ/cm^2^) under a thin layer of PBS, and further cultivated in supplement-free medium in the CO_2_ incubator for 10 hr. Cells were collected for further experiments, including cell apoptosis, cell stress, and cell cycle.

#### 2.8.2. Detection of KCs Apoptosis

The determination of cell apoptosis using Nexin reagent kit was performed as described above in cell apoptosis analysis. Three independent experiments were performed in this study. Each experiment was repeated in triplicate (*n* = 3).

#### 2.8.3. Determination of Oxidative Stress in KCs

Intracellular ROS generation and nitric oxide (NO) activity were determined using the Muse® Oxidative Stress Kit (Merck Millipore, Massachusetts, USA) and Muse® Nitric Oxide Kit (Merck Millipore), respectively. Briefly the collected cells were incubated with superoxide (O_2_^·−^) or NO working solution, according to the manufacturer's instructions. For ROS assay, working solution was prepared by mixing the oxidative stress intermediate solution in 1X assay buffer (ratio 1 : 80). 190 *μ*L of working solution was added to 10 *μ*L of cell suspension. The mixture was incubated at 37°C for 30 min (protect from light). The percentages of cells with negative ROS (−) and positive ROS (+) were measured using a flow cytometer (Muse™ Cell Analyzer, Merck Millipore, Darmstadt, Germany). For NO assay, cells were incubated with nitric oxide reagent at 37°C for 30 min followed by mixing with 7-AAD dye reagent. The NO activity (live and dead cells) was measured using a flow cytometer (Muse™ Cell Analyzer, Merck Millipore, Darmstadt, Germany). Three independent experiments were performed both in ROS and in RNS studies. Each experiment was repeated in triplicate (*n* = 3).

#### 2.8.4. Analysis of Cell Cycle

Cell cycle phases were determined using the Guava® Cell Cycle Kit (Merck Millipore). Briefly, the collected cells were fixed with 70% ethanol. Next, cell pellets were incubated with 200 *μ*L of cell cycle reagent (propidium iodide (PI)), according to the manufacturer's instructions. Cells were incubated for 30 min at room temperature in the dark. The percentages of cells in each cell cycle phase, including G0/G1, S, and G2/M, were measured using flow cytometry. (Muse™ Cell Analyzer, Merck Millipore, Darmstadt, Germany). Three independent experiments were performed in this study. Each experiment was repeated in triplicate (*n* = 3).

#### 2.8.5. Determination of Apoptosis-Related Protein Expression

The apoptosis-related proteins, including caspases-3, 8, and 9, were determined by using Western blotting. The extraction of total proteins from the collected cells was conducted, according to previous studies [27]. The protein content in the samples was determined using a DC^TM^ protein assay kit (Bio-Rad Laboratories, Inc., California, USA), according to the manufacturer's instructions. For Western blot analysis, the sample was loaded on 15% SDS-polyacrylamide gel, and proteins were separated at 60 mA for 90 min. Proteins were transferred to a polyvinylidene difluoride (PVDF) membrane (Pall Corporation, Florida, USA) using the Pierce G2 Fast Blotter (Thermo Fisher Scientific, Rockford, China) at 25 V for 7 min. Transblotted membrane was incubated at room temperature for 2 hr in blocking solution composed of 5% skim milk in Tris-buffered saline with Tween 20 buffer (TBST), washed, and then incubated with primary anti-caspase-8 (1 : 1000), 9 (1 : 500), and 3 (1 : 1000) antibody (ab32397, ab32539, and ab32351, Abcam, Cambridge, UK) or anti-beta-actin (MABT523, Millipore Corp, Temecula, California, USA) at 4°C overnight. Thereafter, transblotted membranes were washed 3 times and incubated with IgG secondary antibody conjugated to horseradish peroxidase (HRP) (1 : 1000) (AP307P, Millipore Corp) at room temperature for 1 hr. Membranes were washed 3 times, and then developed using a chemiluminescence detection kit (Luminata™ Forte Western HRP Substrate, Merck Millipore). Four independent experiments were performed in this study.

#### 2.8.6. Determination of Cyclobutane Pyrimidine Dimers (CPDs) and Pyrimidine-Pyrimidone (6-4) Photoproducts (PPs) Lesions

Both CPDs and 6-4 PPs lesions were determined by using the enzyme-linked immunosorbent assay. The genomic DNA of the collected cells was extracted by using a NucleoSpin column (Macherey-Nagel GmbH & Co. KG, Duren, Germany), according to the manufacturer's instructions. CPDs and 6-4 PPs lesions were measured by using the OxiSelect™ UV-induced DNA Damage ELISA Kit (Cell Biolabs, San Diego, USA). Briefly, 10 hr after UVB exposure, the extracted DNA was converted to single-stranded DNA by heating at 95°C for 10 min and immediately chilling on ice for 10 min. 50 *μ*L of CPDs/6-4 PPs DNA standards or samples was added to the DNA high-binding plate. After that, 50 *μ*L of DNA binding solution was added, mixed, and incubated at room temperature overnight. The DNA solutions were removed. The plate was washed twice with PBS. 200 *μ*L of blocking solution was added and incubated for 1 hr at room temperature. CPDs and 6-4 PPs lesions were probed with mouse monoclonal anti-CPD antibody and mouse monoclonal anti-(6-4) PP (volume and dilution described in the manufacturing detail) for 1 hr at room temperature. Thereafter, the plate was washed 3 times with wash buffer and incubated with HRP-conjugated secondary antibody for 1 hr at room temperature. 100 *μ*L of substrate solution was added and incubated at room temperature for 10 min. 100 *μ*L of stop solution was added immediately after the color changed). Finally, the absorbance was read at 450 nm using a microplate reader. Four independent experiments were performed in this study. Each experiment was repeated in triplicate (*n* = 3).

### 2.9. Statistical Analysis

Data are expressed as mean ± S.D. Three/four independent experiments were performed in this study (*N* ≥ 3). Each experiment was repeated in triplicate (*n* = 3). Student's unpaired *t*-test was used to compare groups, and *p* < 0.05 was considered significant.

## 3. Results

### 3.1. Appearance and Qualification of the NAR

**Figure 1:**
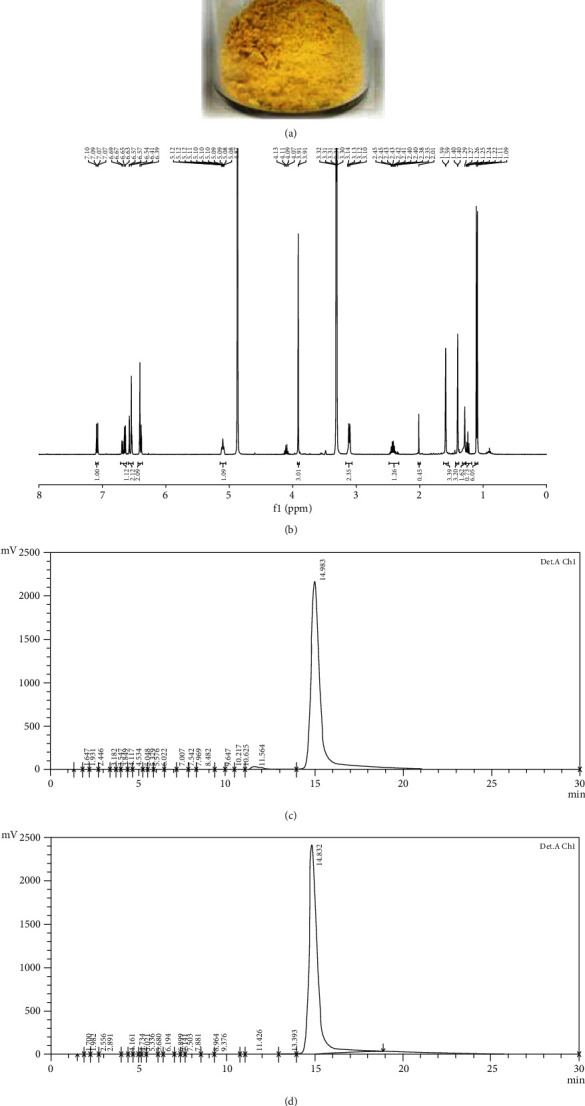
Characterization and qualification of the NAR: (a) the appearance of the NAR, (b) NMR spectrum of the NAR, (c) HPLC chromatogram of the standard artocarpin, and (d) HPLC chromatogram of the NAR.

The obtained NAR is a light-yellow powder (Figure 1(a)). The NMR spectrum of the NAR identified the chemical structure of artocarpin (Figure 1(b)) [27]. The content of artocarpin in the NAR was 99.30 ± 0.03%, according to the HPLC calibration curve generated from the standard artocarpin. The HPLC chromatograms of the standard artocarpin and NAR are shown in Figures 1(c) and 1(d), respectively.

### 3.2. Antilipid Peroxidation Activity of the NAR

The antilipid peroxidation potential of the NAR was determined by TBARS assay and compared to the trolox standard antioxidant. Inhibition percentages of lipid peroxidation of the trolox and the NAR were dose-dependent with IC_50_ values of 7.3 ± 0.8 and 18.2 ± 1.6 *μ*g/mL, respectively. The IC_50_ value of the NAR was greater than that of the standard (data not shown).

### 3.3. Effects of UVB Doses on Human KCs Viability, Morphology, and Apoptosis

**Figure 2:**
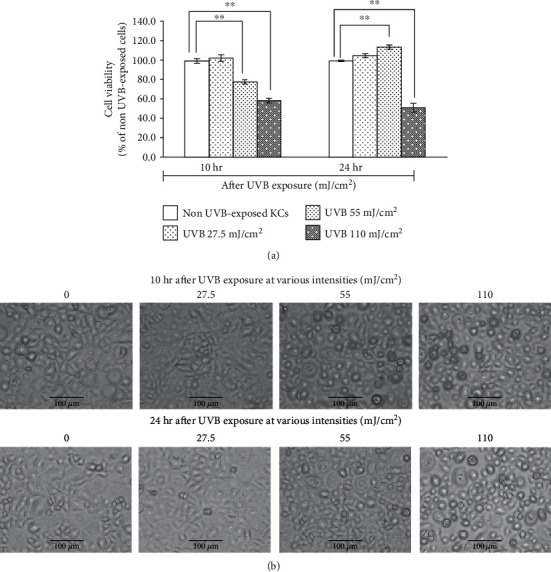
Viability and morphology of human skin keratinocytes (KCs) after UVB exposure at an intensity of 27.5, 55, or 110 mJ/cm^2^. (a) Viability of cells was measured by using the XTT assay, and results were calculated as mean ± S.D.^∗∗^*p* < 0.01, using unpaired Student's *t*-test. (b) Morphology of the non-UVB-exposed cells and the cells cultured for 10 and 24 hr after UVB exposure at various intensities was observed by light microscopy and photographed at a magnification of 200x (scale bar: 100 *μ*m).

The effects of UVB doses (27.5, 55, or 110 mJ/cm^2^) on the viability and morphology of KCs at 10 and 24 hr after irradiation are represented in Figure 2. According to the XTT assay, Figure 2(a) shows the cell viability expressed as the percentages of UVB-exposed cells versus non-UVB-exposed cells, which is normalized to 100%. The viability of cells exposed to UVB at 27.5 mJ/cm^2^ was similar to that of the non-UVB-exposed cells. However, the viability of cell exposed to UVB at higher intensities, 55 and 110 mJ/cm^2^, decreased, respectively, to 78.4 ± 2.1% and 58.6 ± 2.4% at 10 hr postirradiation. The recovery of cell viability to about 100% was seen at 24 hr after exposure at 55 mJ/cm^2^ whereas such recovery was not seen at 110 mJ/cm^2^. The morphology of cells at 10 and 24 hr after UVB exposure at various intensities is shown in Figure 2(b). Non-UVB-exposed cells or cells exposed to a low dose of UVB displayed a typical cuboidal shape of KCs. Cells exposed to UVB at 55 mJ/cm^2^ exhibited at 10 hr postirradiation morphological characteristics of apoptosis, such as shrinking and membrane blebbing. After 24 hr UVB exposure, many cells recovered their morphology, while cells exposed to UVB at 110 mJ/cm^2^ exhibited irreversible hallmarks of both apoptosis and necrosis, including cell shrinkage, disrupted cell membrane, and cell swelling.

**Figure 3:**
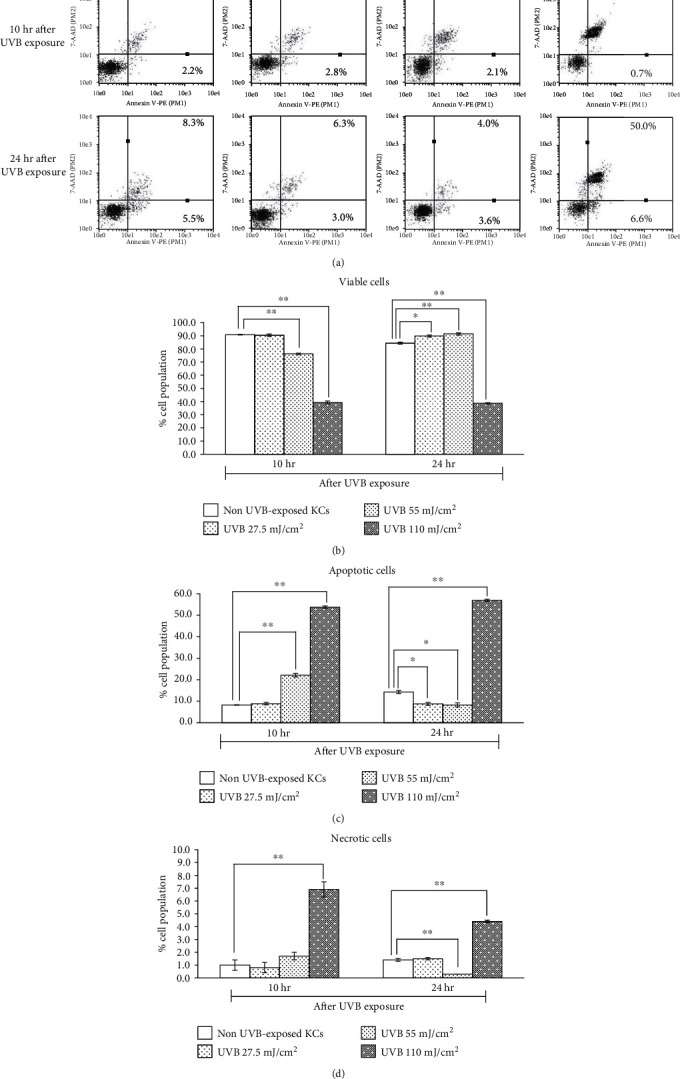
Percentages of viable (VC), total apoptotic (TA), and necrotic (NC) human skin KCs after UVB exposure at 25.7, 55, and 110 mJ/cm^2^. (a) Examples of representative dot plots of Annexin V-PE/7-AAD analyzed by flow cytometry and bar graphs indicating percentages of (b) VC, (c) TA, and (d) NC at 10 and 24 hr after UVB exposure. ^∗^*p* < 0.05 and ^∗∗^*p* < 0.01, using unpaired Student's *t*-test.

According to apoptotic detection by using Annexin V/7-AAD staining, percentages of viable cells (VC), total apoptotic cells (TA), and necrotic cells (NC) at 10 and 24 hr incubation after UVB exposure are shown in Figure 3(a) and Table S1. Viability results were quite similar to the XTT assay. Increasing UVB dose resulted in decreased cell viability, particularly at 10 hr after UVB exposure (73.9 ± 0.8% for 55 mJ/cm^2^ and 39.3 ± 1.1% for 110 mJ/cm^2^ UVB intensity). At 24 hr incubation after UVB exposure, cells exposed to 55 mJ/cm^2^ restored 91.4 ± 0.9% viability whereas cells exposed to 110 mJ/cm^2^ remained 38.7 ± 0.5 viable. Significant induction of apoptosis was found in cells exposed to 55 mJ/cm^2^ (22.1 ± 0.8) at 10 hr after UVB exposure. At 10 hr and 24 hr after UVB exposure, the highest UVB dose increased the percentages of both TA (Figure 3(b)) and NC (Figure 3(c)) and decreased the percentages of VC (Figure 3(d)) compared to the non-UVB-exposed cells. Data showed that the lowest UVB dose did not affect cells whereas the highest dose induced irreversible detrimental effect for KCs. Therefore, exposure to UVB at 55 mJ/cm^2^ exerted no excessive cell death, confirming that the dose was selected for all the assays of the NAR.

### 3.4. Protective Effects of the NAR against UVB-Induced Human KCs Apoptosis

**Figure 4:**
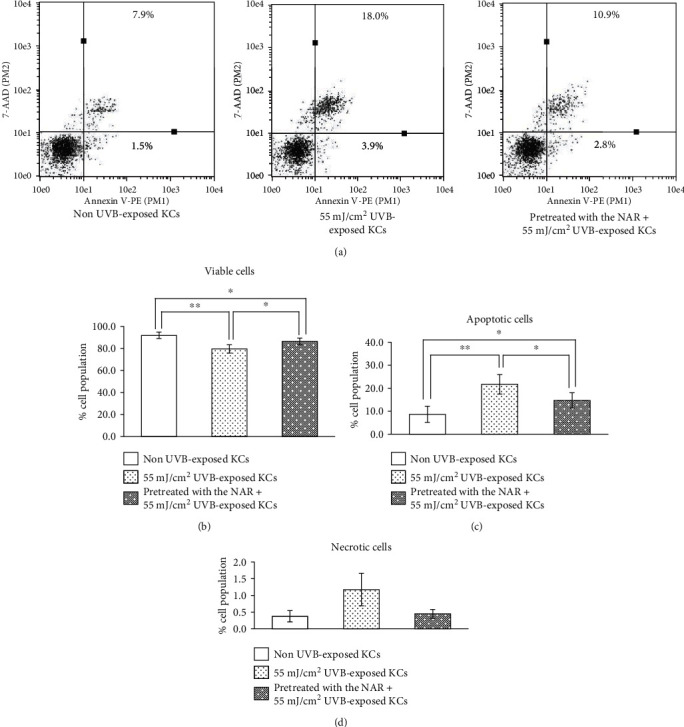
Percentages of VC, TA, and NC of human skin KCs unpretreated or pretreated with 3.1 *μ*g/mL of NAR for 24 hr, exposed with UVB at the intensity of 55 mJ/cm^2^, and then collected at 10 hr after UVB exposure. (a) Examples of representative dot plots of Annexin V-PE/7-AAD analyzed by flow cytometry. Bar graphs indicating percentages of (b) VC, (c) TA, and (d) NC cells. ^∗^*p* < 0.05 and ^∗∗^*p* < 0.01, using unpaired Student's *t*-test.

To evaluate the protective effects of the NAR, cells were pretreated with the extract before UVB exposure. Percentages of VC, TA, and NC are shown in Table S2. UVB exposure increased the percentages of apoptotic cells. Pretreatment with the NAR decreased the percentages of UVB-induced apoptotic cells (13.1 ± 2.9) compared to UVB-exposed cells (19.3 ± 3.7), as shown in Figure 4.

### 3.5. Reduction by the NAR of ROS Overproduction in UVB-Exposed KCs

**Figure 5:**
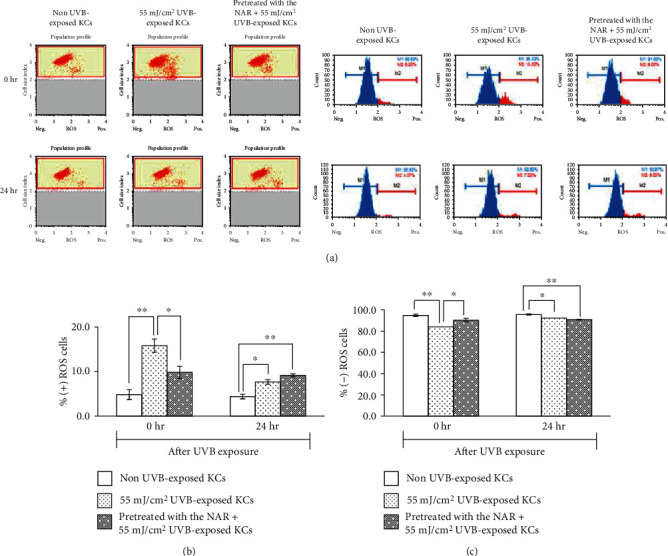
Percentages of positive ROS ((+) ROS) and negative ROS ((-) ROS) human skin KCs unpretreated or pretreated with 3.1 *μ*g/mL of NAR for 24 hr, exposed with UVB at the intensity of 55 mJ/cm^2^, and then collected immediately (0 hr) and 24 hr after UVB exposure. (a) Examples of cell population profiles in both dot plots and chromatograms. (b) Bar graphs indicating percentages of (b) ROS-positive cells and (c) ROS-negative cells. ^∗^*p* < 0.05 and ^∗∗^*p* < 0.01, using unpaired Student's *t*-test.

In the next step, we determined the effects of the NAR against UVB-induced overproduction of ROS (oxidative stress) in human KCs. As we have known, ROS act as a mediator of UVB-induced apoptosis. Intracellular ROS were detected by the fluorescence intensity of a dihydroethidium (DHE) probe and measured by both flow cytometry representative dot plots and chromatogram profiles (Figure 5(a)). We observed that ROS levels increase immediately after UVB exposure. Pretreatment with the NAR greatly reduced the percentages of ROS-positive cells (9.8 ± 1.4), compared to nontreated irradiated cells (15.8 ± 1.5), at the early time point after UV exposure (Figures 5(b) and 5(c), Table S3). No significant differences were observed in NAR-pretreated + UVB-exposed cells and UVB-exposed cells at 24 hr after UV exposure.

### 3.6. Suppression by the NAR of NO Generation in UVB-Exposed KCs

**Figure 6:**
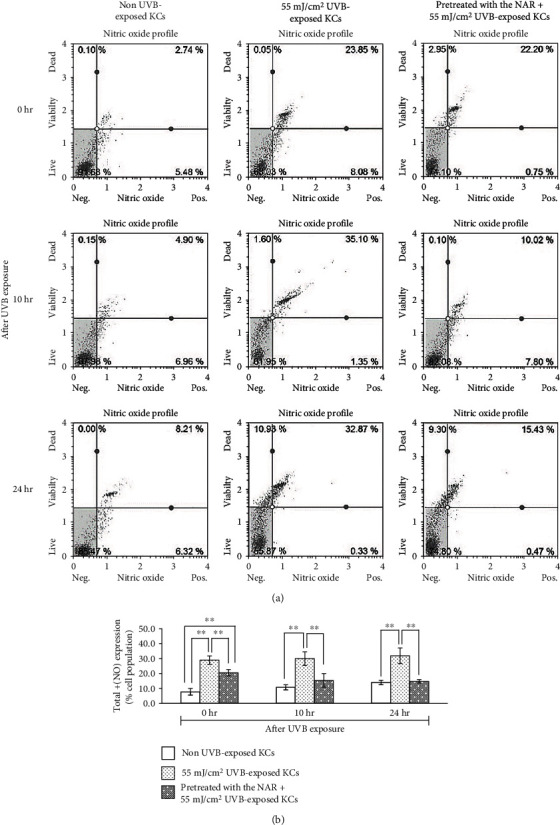
Effects of UVB-induced intracellular NO accumulation in human KCs. Cells were pretreated with the NAR for 24 hr and exposed to UVB. At different time points (0, 10, and 24 hr), total NO formation was measured using a flow cytometer. (a) Examples of dot plot profiles. The lower left quadrant (NO-/7-AAD-) means viable cells. The lower right quadrant (NO+/7-AAD-) shows viable cells with NO generation. The upper right quadrant (NO+/7-AAD+) shows cell death with NO formation. The upper left quadrant (NO-/7-AAD+) shows cell death without NO production. (b) Bar graphs show the percentage of NO-positive KCs in total cells. ^∗∗^*p* < 0.01, using unpaired Student's *t*-test.

Not only ROS-mediated apoptosis in human KCs but also reactive nitrogen species (RNS) including NO-mediated apoptosis were further investigated. To determine the protective effects of the NAR against NO formation in UVB-exposed cells, intracellular NO was detected using a NO fluorogenic probe after pretreatment with the extract and UVB exposure. Representative dot plots of flow cytometry are shown in Figure 6(a). The percentages of NO-positive KCs in total cell population (live + dead) (Table S4) were significantly higher in UVB-exposed cells compared to non UVB-exposed cells at various time points (Figure 6(b)), while pretreatment with the NAR surprisingly led to a continuously significant decrease of the percentage of NO-positive KCs in the total cell population at 0 (-8.5%), 10 (-14.6%) and 24 hr (-17.1%) after UVB exposure, compared to UVB-exposed cells.

### 3.7. Cell Cycle Responses of UVB-Exposed Cells to the NAR

**Figure 7:**
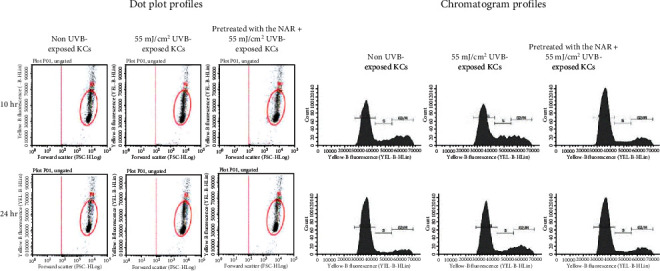
Cell cycle phases of human skin KCs unpretreated or pretreated with 3.1 *μ*g/mL of NAR for 24 hr, exposed with UVB at the intensity of 55 mJ/cm^2^, and then collected at 10 and 24 hr after UVB exposure. This figure shows the examples of cell cycle distribution in dot plots and histogram profiles.

Figure 7 illustrates representative flow cytometry dot plots and chromatogram profiles of cell cycle. Table S5 shows the percentages of cells in the cell cycle phases (G0/G1, S, and G2/M). Results revealed that UVB exposure induces a decrease of the percentages of cells in the G0/G1 phase and an increase of the percentages of cells in the S phase. Although the G2/M phase was not significantly affected at 10 hr post-UVB exposure, an increase was observed at 24 hr post-UVB exposure. Interestingly, pretreatment of UVB-exposed cells to the NAR resulted in an accumulation of cells in the G0/G1 phase (+9% and +7.6% at 10 and 24 hr postexposure, respectively), concomitant with decreases of cells in the S phase (-6.5% and -3.1% at 10 and 24 hr postexposure, respectively) and G2/M phase (-4.3% at 24 hr post-UVB exposure).

### 3.8. Modulation by the NAR of Apoptosis-Related Protein (Caspases) Production in UVB-Exposed KCs

**Figure 8:**
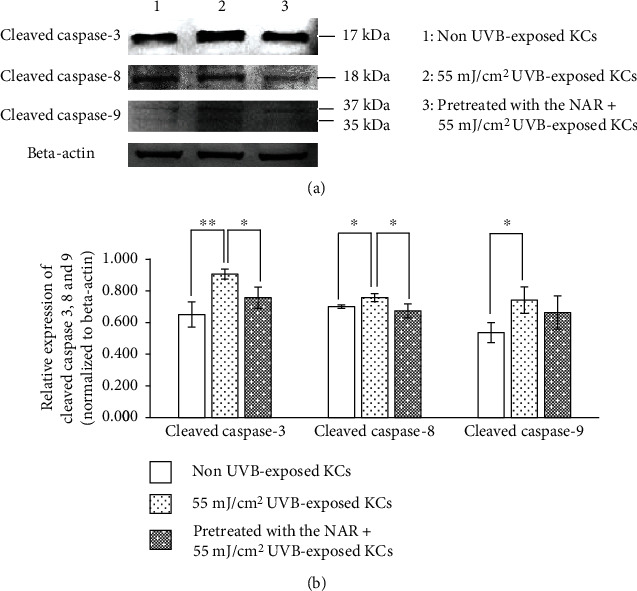
Apoptosis-related protein expression in human skin KCs unpretreated or pretreated with 3.1 *μ*g/mL of NAR for 24 hr, exposed with UVB at the intensity of 55 mJ/cm^2^, and then collected at 10 hr after UVB exposure. (a) Cleaved caspase (3, 8, and 9) levels were determined by Western blotting of whole cell lysates (b) Bar graphs show the relative expression of the cleaved caspase proteins that normalized to beta-actin. Data are expressed as the mean ± S.D.^∗^*p* < 0.05 and ^∗∗^*p* < 0.01, using unpaired Student's *t*-test.

The effect of the NAR on the expression of caspases was determined using Western blot analysis. We studied the expression of both initiator caspases-8 and 9 because they are involved in the extrinsic and intrinsic apoptosis pathways, respectively. In addition, we assessed the expression of caspase-3 which plays a central role in apoptosis. We analyzed these proteins after 10 hr of UVB exposure. Cleaved caspase-3, 8, and 9 bands were detected (Figure 8(a)). Our study showed that UVB-exposed cells significantly upregulated the expression of cleaved caspases-3, 8, and 9, compared to non UVB-exposed cells (Figure 8(b)). Treatment of KCs with the NAR for 24 hr prior to UVB irradiation resulted in a decrease in the expression of cleaved caspases-3 and 8. While the NAR pretreatment did not change the cleaved caspase-9 expression significantly.

### 3.9. Protective Effects of the NAR on DNA Lesions Induced by UVB in Human KCs

**Figure 9:**
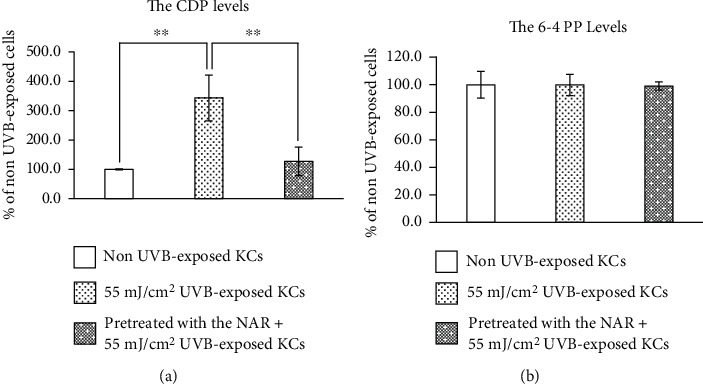
(a) Cyclobutane pyrimidine dimers (CPDs) and (b) pyrimidine-pyrimidone (6–4) photoproduct (6–4 PPs) formation in human skin KCs unpretreated or pretreated with 3.1 *μ*g/mL of NAR for 24 hr, exposed to UVB at the intensity of 55 mJ/cm^2^, and then collected at 10 hr after UVB exposure. Data are expressed as the mean ± S.D.^∗∗^*p* < 0.01, using unpaired Student's *t*-test.

We analyzed the effect of UVB-induced DNA lesions, both CPDs and 6-4 PPs lesions, in human KCs by an enzyme immunoassay. As shown in Figure 9(a), we observed that CPDs is the dominant DNA lesion in KCs after UVB irradiation. An approximately 3-fold increase in CPDs formation was found from our experiments, compared to non UVB-exposed cells, which corroborates the findings of the previous reports [3, 28]. UVB exposure did not significantly change the levels of 6-4 PP lesions (Figure 9(b)). Pretreatment with the NAR greatly prevented UVB-induced CPDs formation in human KCs.

## 4. Discussion

Climate change and depletion of the ozone layer may lead to an increase of the ground level of solar radiation, with more intense surface UVB radiation globally [29]. Our skin endogenous defense mechanisms against UV irradiation (antioxidant and DNA repair systems) can be overwhelmed by excessive UVB exposure. Overexposure to UVB leads to cellular damages in the epidermis including alteration in the structure of protein, DNA, lipid membrane, and many other biologically important molecules. Ultimately, these oxidative damages cause several pathological changes such as sunburn, inflammation, hyperplasia, skin aging, and skin cancer [6, 18]. Traditional medicine and natural products may help to prevent and reduce these harmful effects of UV [30]. Plant-derived photoprotective agents with UV absorption, antioxidation, and anti-inflammation properties have great interest to be used as natural photoprotectors and to be free from side effects of synthetic chemical-derived photoprotective agents [28, 31, 32]. The natural artocarpin (NAR), a prenylated flavonoid, is a major compound isolated from *Artocarpus altilis* plant. Many researches have tested the pharmacological activities of *A. altilis*. Artocarpin possesses several biological effects including antioxidant activity, depending on the dose [6, 18–22]. The antioxidant activity of phenolic compounds which include flavonoids is generally determined by their chemical structure and their ability to scavenge hydroxyl radicals. In this study, the NAR extract was screened for its antilipid peroxidation activity, utilizing the TBARS method. Overproduction of ROS rapidly interacts with unsaturated fatty acids of the organelle membrane and induces lipid peroxidation. The oxidation of lipid-by-lipid peroxyl radicals (ROO^·^) occurs as a chain reaction and finally causes defective membranes and cell death. MDA which is the final product of lipid peroxidation can activate the mitogen-activated protein kinase (MAPK) signalling pathway and ultimately leads to the induction of caspase activation and cell death [33, 34]. In this study, the NAR offered a weaker antilipid peroxidation activity (higher IC_50_) than trolox. Trolox is composed of both lipophilic and lipophobic groups [35, 36], while the NAR is a lipophilic phenolic antioxidant that displays intermediate or slow reaction rate [37]. We speculate that the NAR slowly reacts with lipid peroxyl radicals in the lipid part and is less effective to inhibit the peroxyl radicals in the aqueous part.

Many evidences suggested that artocarpin extracted from *A. altilis* plant has photoprotective effects on UVB-induced skin pathological changes including inflammation (TNF-*α*, IL-1*β*, and IL-6), desquamation, and epidermal thickening [18, 22]. Keratinocytes (KCs) apoptosis is a main marker for UVB exposure-induced skin damage. There have been reports that UVB-induced KCs apoptosis involves DNA damage, cell surface death receptor activation, and free radical formation [2, 8–10]. The prevention or reduction of these events may be played by active components with UV-absorber, antioxidation, antilipid peroxidation, anti-inflammatory, and antiapoptotic properties [3, 32, 38]. There has been no study on the photoprotective effects of the NAR on UVB-induced apoptosis in KCs. In the present study, we pretreated human KCs with the NAR before UVB exposure and then observed its ability to protect or attenuate the several effects of UVB leading to KCs apoptosis. Our experiments used an intermediate dose of UVB (55 mJ/cm^2^) to induce cell apoptosis with altered morphology. We revealed that the NAR attenuates human KCs apoptosis induced by UVB. UV-induced apoptosis is a complex process involving several pathways like generation of ROS (O_2_^·-^, ^·^OH, and H_2_O_2_) and RNS (nitric oxide (^·^NO)) in high amounts, activation of cell surface death receptors, and induction of genomic DNA damages. There has been a report that at least 50% of the UVB-induced cellular damages result from the increased level of ROS [28]. As we have known, massive ROS not only cause oxidative damage to lipid cell membranes, nucleic acids, and mitochondria organelles but also modulate transduction signals involving the activation of cell surface death receptors (TNF-R1, Fas), and the activation of p53/Bax (proapoptotic protein) and/or MAP kinases (JNK and p38 kinase) through the intrinsic pathway [9, 11, 14]. UVB exposure induces also the production of ^·^NO which plays a role in the regulation of cell apoptosis. ^·^NO can rapidly react with superoxide radicals (O_2_^·-^) to produce peroxynitrite (ONOO^−^) which is a powerful oxidative molecule and potent inducer of apoptosis [13, 14]. In this study, the level of ^·^NO was completely suppressed to the basal level (non-UVB-exposed cells) at 24 hr after UVB exposure. A positive correlation was found between the antioxidant capacity of the NAR and its nitric oxide scavenging activity. Therefore, we hypothesized that the NAR prevents UV-induced KCs apoptosis via trapping O_2_^·-^ and ^·^NO and regulating the downstream apoptotic signalling components such as MAPKs, cytochrome C, and caspases [8].

UV-induced KCs apoptosis is a complex mechanism in which many molecular pathways are related. The effect of UVB-induced DNA damage is the notable event of UVB-induced cell death [10]. These damages can lead to mutations that ultimately may cause skin cancer. Absorption of UVB rays by DNA generates both the formation of CPDs and 6-4 PPs [12, 39]. At 10 hr after UV treatment, our results showed that UVB radiation leads to a large excess of CPDs but does not induce a significant change in the 6-4 PPs level. We suggested that the efficiency of repairing 6-4 PPs is higher than that of CPDs. Previous reports indicated that after 6-4 PPs are initially produced, some are repaired and removed by 6 hr [40]. It was also noted that 6-4 PPs are quickly repaired and produced lesser than CPDs [17]. In addition, our data clearly demonstrated that the NAR pretreatment decreases the CPD level in UVB-irradiated KCs. We theorized that the NAR may decrease CPD lesion formation via the UVB absorber property [6]. The same as many studies, we used a UVB dose which results in the formation of apoptotic KCs and cell cycle arrest. Our results showed that 10 hr after UVB exposure, cells are unable to move onto the G2/M phase and accumulate in the S phase. Our intermediate UV dose induced a low level of apoptotic KCs with 22% of cell population. The UV-induced DNA damage was excessive and was not efficiently repaired, leading to activation of the apoptotic pathway, which eliminates severely damaged cells. At 24 hr after UVB, surviving cells were capable to progress their cell cycle, despite a certain delay. Less than 10% of the cell population were apoptotic KCs. The tumor suppressor protein p53 is essential in multiple cellular processes, cell cycle arrest for repairing DNA damage, and finally causing cysteine protease (caspases) activation and apoptosis [17]. We investigated the activation of a group of caspases, upstream initiators such as caspases-8 (the extrinsic pathway) and 9 (the intrinsic pathway), and downstream effectors including caspase-3 [15]. Caspases are initially produced as inactive monomeric procaspases that require dimerization and often cleavage for activation. UV-induced apoptosis phenomenon was observed; our data showed that 10 hr after UVB exposure, the expression of cleaved caspases-3, 8, and 9 is increased. We suggested that caspase-3 activity is not induced until 10 hr after UV exposure and caspase-8 activity does not precede caspase-3 activity. The pretreatment with the NAR increased the number of UV-irradiated KCs in the G0/G1 phase over 24 hr and decreased the number of UV-induced apoptotic KCs. In addition, it reduced the expression of cleaved caspases-3 and 8. The pretreatment with the NAR prevented UVB-induced skin damage such as DNA lesions through cell cycle arrest at the G0/G1 phase. This growth arrest allowed cells to repair DNA damages. The pretreatment with the NAR contributed also to reduce UVB-induced apoptosis by modulating several apoptotic pathways. It seemed probable that the photoprotective effect of the NAR is p53-dependent and mediated through the Bax and caspase-3 pathway. Considering that apoptosis mediated by the p53/p21/Bax/bcl-2 pathway and epidermal hyperplasia are linked, the NAR was likely to reduce basal cell hyperproliferation. Activation of cell surface death receptors with their ligands (such as TNF-*α*) is also a crucial event in UVB-mediated apoptosis. UVB irradiation induces the release of the proinflammatory cytokine TNF-*α* which initiates both inflammation and apoptosis [16]. Our previous study reported that artocarpin suppresses TNF-*α* release in UVB-irradiated KCs [18]. This implied that the NAR may eventually decrease apoptosis via the downstream regulation of the TNF-*α*/caspase-8/caspase-3 pathway. Therefore, the NAR showed a photoprotective effect against UVB-induced apoptosis. Nevertheless, the deeper molecular mechanism of its action needs further investigation.

## 5. Conclusion

**Figure 10:**
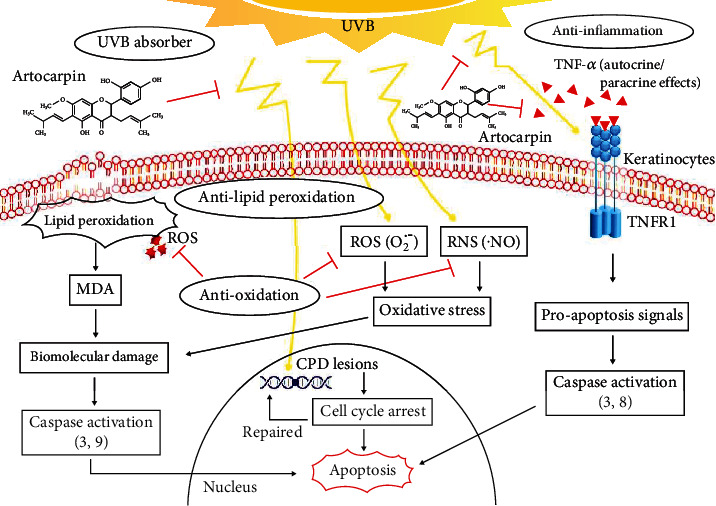
Schematic summary of the photoprotective effects of the natural artocarpin (NAR). The NAR protects the deleterious effects of UVB-induced KCs apoptosis by UV-absorber (protects DNA damage and cell surface death receptor activation), antioxidation (protects macromolecule damage), antilipid peroxidation (protects organelle membrane damage), and anti-inflammatory (modulates TNF*α* production (complementary information from a previous study on the same extract)) effects.

The present data indicate that the NAR protects human KCs from the deleterious effects of UVB radiation causing KCs apoptosis by the following properties: UV absorber, DNA protector, antilipid peroxidation, and antioxidation (see Figure 10). They provide complementary information on artocarpin that had previously been studied for its anti-inflammatory property [18]. The extract may protect skin by modulating UVB-induced apoptotic signalling cascades: (i) DNA lesion (CPD)/caspase activation (9 and 3)/apoptosis, (ii) lipid peroxidation (MDA)/caspase activation (9 and 3)/apoptosis, (iii) free radical formation/damaged macromolecules/caspase activation/apoptosis, and (iv) TNF-*α* ligand/TNF receptor activation/caspase activation (8 and 3)/apoptosis. Thus, the NAR may play a role in the prevention of UVB-induced skin damage including epidermal hyperplasia. This work suggests that the NAR is suitable for photoprotective formulations and skin care products.

## Data Availability

The data used to support the findings of this study are available from the corresponding author upon request.

## Supplementary Materials

Supplementary Materials Supplementary Table S1: the percentages of viable (VC), total apoptotic (TA), and necrotic (NC) human skin KCs after UVB exposure. Supplementary Table S2: the percentages of VC, TA, and NC of human skin KCs unpretreated or pretreated with the extract and exposed with UVB. Supplementary Table S3: the percentages of positive ROS and negative ROS human skin KCs unpretreated or pretreated with the extract and exposed with UVB. Supplementary Table S4: the percentages of total NO-positive human KCs in a cell population. Supplementary Table S5: the percentages of cell cycle distribution of unpretreated or pretreated with the extract and exposed with UVB

## References

[1] Fadeel B., Orrenius S., Zhivotovsky B. (1999). Apoptosis in human disease: a new skin for the old ceremony?. *Biochemical and Biophysical Research Communications*.

[2] Reefman E., Limburg P. C., Kallenberg C. G. M., Bijl M. (2005). Apoptosis in human skin: role in pathogenesis of various diseases and relevance for therapy. *Annals of the New York Academy of Sciences*.

[3] Calo R., Marabini L. (2014). Protective effect of *Vaccinium myrtillus* extract against UVA- and UVB-induced damage in a human keratinocyte cell line (HaCaT cells). *The Journal of Photochemistry and Photobiology B*.

[4] Panich U., Sittithumcharee G., Rathviboon N., Jirawatnotai S. (2016). Ultraviolet radiation-induced skin aging: the role of DNA damage and oxidative stress in epidermal stem cell damage mediated skin aging. *Stem Cells International*.

[5] Dunaway S., Odin R., Zhou L., Ji L., Zhang Y., Kadekaro A. L. (2018). Natural antioxidants: multiple mechanisms to protect skin from solar radiation. *Frontiers in Pharmacology*.

[6] Lee C.-W., Ko H.-H., Chai C.-Y., Chen W.-T., Lin C.-C., Yen F.-L. (2013). Effect of *Artocarpus communis* extract on UVB irradiation-induced oxidative stress and inflammation in hairless mice. *International Journal of Molecular Sciences*.

[7] Murphy G., Young A. R., Wulf H. C., Kulms D., Schwarz T. (2001). The molecular determinants of sunburn cell formation. *Experimental Dermatology*.

[8] Assefa Z., Van Laethem A., Garmyn M., Agostinis P. (2005). Ultraviolet radiation-induced apoptosis in keratinocytes: on the role of cytosolic factors. *Biochimica et Biophysica Acta (BBA) - Reviews on Cancer*.

[9] Ryu H.-C., Kim C., Kim J.-Y., Chung J.-H., Kim J.-H. (2010). UVB radiation induces apoptosis in keratinocytes by activating a pathway linked to “BLT2-reactive oxygen species”. *Journal of Investigative Dermatology*.

[10] Kulms D., Zeise E., Poppelmann B., Schwarz T. (2002). DNA damage, death receptor activation and reactive oxygen species contribute to ultraviolet radiation-induced apoptosis in an essential and independent way. *Oncogene*.

[11] Fridman J. S., Lowe S. W. (2003). Control of apoptosis by p53. *Oncogene*.

[12] Matsumura Y., Ananthaswamy H. N. (2004). Toxic effects of ultraviolet radiation on the skin. *Toxicology and Applied Pharmacology*.

[13] Snyder C. M., Shroff E. H., Liu J., Chandel N. S. (2009). Nitric oxide induces cell death by regulating anti-apoptotic BCL-2 family members. *PLoS One*.

[14] Redza-Dutordoir M., Averill-Bates D. A. (2016). Activation of apoptosis signalling pathways by reactive oxygen species. *Biochimica et Biophysica Acta (BBA) - Molecular Cell Research*.

[15] Elmore S. (2016). Apoptosis: a review of programmed cell death. *Toxicologic Pathology*.

[16] Yoshizumi M., Nakamura T., Kato M. (2008). Release of cytokines/chemokines and cell death in UVB-irradiated human keratinocytes, HaCaT. *Cell Biology International*.

[17] Sabio G., Davis R. J. (2014). TNF and MAP kinase signalling pathways. *Seminars in Immunology*.

[18] Tiraravesit N., Yakaew S., Rukchay R. (2015). *Artocarpus altilis* heartwood extract protects skin against UVB in vitro and in vivo. *The Journal of Ethnopharmacology*.

[19] Donsing P., Limpeanchob N., Viyoch J. (2008). Evaluation of the effect of Thai breadfruit's heartwood extract on melanogenesis-inhibitory and antioxidation activities. *International Journal of Cosmetic Science*.

[20] Itsarasook K., Ingkaninan K., Viyoch J. (2014). Artocarpin-enriched extract reverses collagen metabolism in UV-exposed fibroblasts. *Biologia*.

[21] Lan W. C., Tzeng C. W., Lin C. C., Yen F. L., Ko H. H. (2013). Prenylated flavonoids from *Artocarpus altilis*: antioxidant activities and inhibitory effects on melanin production. *Phytochemistry*.

[22] Lee C. W., Ko H. H., Lin C. C., Chai C. Y., Chen W. T., Yen F. L. (2013). Artocarpin attenuates ultraviolet B-induced skin damage in hairless mice by antioxidant and anti-inflammatory effect. *Food and Chemical Toxicology*.

[23] Buranajaree S., Donsing P., Jeenapongsa R., Viyoch J. (2011). Depigmenting action of a nanoemulsion containing heartwood extract *of Artocarpus incisus* on UVB-induced hyperpigmentation in C57BL/6 mice. *International Journal of Cosmetic Science*.

[24] Ohkawa H., Ohishi N., Yagi K. (1979). Assay for lipid peroxides in animal tissues by thiobarbituric acid reaction. *Analytical Biochemistry*.

[25] Viyoch J., Buranajaree S., Grandmottet F. (2010). Evaluation of the effect of Thai breadfruit’s heartwood extract on the biological functions of fibroblasts from wrinkles. *International Journal of Cosmetic Science*.

[26] Eisinger M., Marko O. (1982). Selective proliferation of normal human melanocytes in vitro in the presence of phorbol ester and cholera toxin. *Proceedings of the National Academy of Sciences*.

[27] Musthapa I., Hakim E. H., Syah Y. M., Juliawaty L. D. (2016). Cytotoxic activities of prenylated flavonoids from *Artocarpus heterophyllus*. *Journal of Engineering and Applied Sciences*.

[28] Wölfle U., Esser P. R., Simon-Haarhaus B., Martin S. F., Lademann J., Schempp C. M. (2011). UVB-induced DNA damage, generation of reactive oxygen species, and inflammation are effectively attenuated by the flavonoid luteolin in vitro and in vivo. *Free Radical Biology and Medicine*.

[29] Bais A. F., Bernhard G., McKenzie R. L. (2019). Ozone–climate interactions and effects on solar ultraviolet radiation. *Photochemical & Photobiological Sciences*.

[30] Markiewicz E., Idowu O. C. (2019). DNA damage in human skin and the capacities of natural compounds to modulate the bystander signalling. *Open Biology*.

[31] Nunes A. R., Vieira Í. G. P., Queiroz D. B. (2018). Use of flavonoids and cinnamates, the main photoprotectors with natural origin. *Advances in Pharmacological and Pharmaceutical Sciences*.

[32] Virag P., Brie I. C., Burz C. C. (2015). Modulation of the UV-B-induced oxidative stress and apoptosis in HaCaT cell line with *Calluna vulgaris* extract. *Notulae Botanicae Horti Agrobotanici Cluj-Napoca*.

[33] Ayala A., Muñoz M. F., Argüelles S. (2014). Lipid peroxidation: production, metabolism, and signaling mechanisms of malondialdehyde and 4-hydroxy-2-nonenal. *Oxidative Medicine and Cellular Longevity*.

[34] Su L.-J., Zhang J.-H., Gomez H. (2019). Reactive oxygen species-induced lipid peroxidation in apoptosis, autophagy, and ferroptosis. *Oxidative Medicine and Cellular Longevity*.

[35] Lucio M., Nunes C., Gaspar D., Ferreira H., Lima J. L. F. C., Reis S. (2009). Antioxidant activity of vitamin E and trolox: understanding of the factors that govern lipid peroxidation studies in vitro. *Food Biophysics*.

[36] Halliwell B. (2011). Free radicals and antioxidants - quo vadis?. *Trends in Pharmacological Sciences*.

[37] Lee J., Cho J. Y., Lee S. Y., Lee K. W., Lee J., Song J. Y. (2014). Vanillin protects human keratinocyte stem cells against ultraviolet B irradiation. *Food and Chemical Toxicology*.

[38] Hamad I., Arda N., Pekmez M., Karaer S., Temizkan G. (2010). Intracellular scavenging activity of Trolox (6-hydroxy-2,5,7,8-tetramethylchromane-2-carboxylic acid) in the fission yeast, Schizosaccharomyces pombe. *Journal of Natural Science, Biology and Medicine*.

[39] Tornaletti S., Pfeifer G. P. (1996). UV damage and repair mechanisms in mammalian cells. *BioEssays*.

[40] Mitchell D. L., Nairn R. S. (1989). The biology of the (6-4) photoproduct. *Photochemistry and Photobiology*.

