# Supplementary material for: Photoprotective Potential of the Natural Artocarpin against In Vitro UVB-Induced Apoptosis
Source: Oxid Med Cell Longev. 2020 Sep 19;2020:1042451. doi: 10.1155/2020/1042451 (PMC7520682; doi:10.1155/2020/1042451)
Supplement: Supplementary Materials — Supplementary Table S1: the percentages of viable (VC), total apoptotic (TA), and necrotic (NC) human skin KCs after UVB exposure. Supplementary Table S2: the percentages of VC, TA, and NC of human skin KCs unpretreated or pretreated with the extract and exposed with UVB. Supplementary Table S3: the percentages of positive ROS and negative ROS human skin KCs unpretreated or pretreated with the extract and exposed with UVB. Supplementary Table S4: the percentages of total NO-positive human KCs in a cell population. Supplementary Table S5: the percentages of cell cycle distribution of unpretreated or pretreated with the extract and exposed with UVB [file 1042451.f1.docx]

# Photoprotective potential of the natural artocarpin against in vitro UVB-induced apoptosis

Kunlathida Luangpraditkun,^1^ Pensri Charoensit,^1^ François Grandmottet,^2^ Céline Viennet,^3^ Jarupa Viyoch,^1^

^1^ Department of Pharmaceutical Technology, Faculty of Pharmaceutical Sciences and Center of Excellence for Innovation in Chemistry, Naresuan University, Phitsanulok, 65000, Thailand

^2^ Department of Biochemistry, Faculty of Medical Science, Naresuan University, Phitsanulok, 65000, Thailand

^3^ UMR 1098 RIGHT INSERM EFS BFC, University of Bourgogne Franche-Comté, Besançon, 25000, France

Correspondences should be addressed to Céline Viennet; [celine.viennet@univ-fcomte.fr](mailto:celine.viennet@univ-fcomte.fr)

Jarupa Viyoch; [jarupav@nu.ac.th](mailto:jarupav@nu.ac.th)

**Supplementary Data**

**Photoprotective potential of the natural artocarpin against in vitro UVB-induced apoptosis**

**Supplementary Tables**

Table S1 Percentages of viable (VC), total apoptotic (TA) and necrotic (NC) human skin KCs after UVB exposure at 25.7, 55 and 110 mJ/cm^2^ were represented as the mean ± S.D. of three independent experiments.

| % Cell  Population | Non UVB-exposed KCs | | 27.5 mJ/cm^2^ UVB-exposed KCs | | 55 mJ/cm^2^ UVB-  exposed KCs | | 110 mJ/cm^2^ UVB-exposed KCs | |
| --- | --- | --- | --- | --- | --- | --- | --- | --- |
|  | 10 hr | 24 hr | 10 hr | 24 hr | 10 hr | 24 hr | 10 hr | 24 hr |
| %VC ± S.D. | 90.3 ± 0.3 | 84.4 ± 0.8 | 90.4 ± 0.9 | 89.8 ± 0.8 | 76.2 ± 0.6 | 91.4 ± 0.9 | 39.3 ± 1.1 | 38.7 ± 0.5 |
| % TA ± S.D. | 8.3 ± 0.1 | 14.3 ± 0.7 | 8.9 ± 0.6 | 8.8 ± 0.7 | 22.1 ± 0.8 | 8.3 ± 1.0 | 53.8 ± 0.6 | 57.0 ± 0.5 |
| % NC ± S.D. | 1.0 ± 0.4 | 1.4 ± 0.1 | 0.8 ± 0.4 | 1.5 ± 0.1 | 1.7 ± 0.3 | 0.3 ± 0.0 | 6.9 ± 0.6 | 4.4 ± 0.1 |

Table S2 Percentages of VC, TA and NC of human skin KCs unpretreated or pretreated with 3.1 µg/mL of NAR for 24 hr, exposed with UVB at the intensity of 55 mJ/cm^2^, and then collected at 10 hr after UVB exposure. Percentages of VC, TA and NC were represented as the mean ± S.D. of three independent experiments.

| % Cell Population | Non UVB-exposed KCs | 55 mJ/cm^2^ UVB-exposed KCs | Pretreated with the NAR +  55 mJ/cm^2^ UVB -exposed KCs |
| --- | --- | --- | --- |
| % VC ± S.D. | 91.9 ± 3.0 | 79.6 ± 3.8 | 86.5 ± 3.0 |
| % TA ± S.D. | 7.7 ± 3.1 | 19.3 ± 3.7 | 13.1 ± 2.9 |
| % NC ± S.D. | 0.4 ± 0.2 | 1.2 ± 0.5 | 0.5 ± 0.1 |

Table S3 Percentages of positive ROS ((+) ROS) and negative ROS ((-) ROS) human skin KCs unpretreated or pretreated with 3.1 µg/mL of NAR for 24 hr, exposed with UVB at the intensity of 55 mJ/cm^2^, and then collected at immediately (0 hr) and 24 hr after UVB exposure. Percentages of cell population with (+) ROS and (-) ROS expression were represented as the mean ± S.D. of three independent experiments.

| Time | Non UVB-exposed KCs | | 55 mJ/cm^2^ UVB -exposed KCs | | Pretreated with the NAR +  55 mJ/cm^2^ UVB -exposed KCs | |
| --- | --- | --- | --- | --- | --- | --- |
|  | % (+) ROS ± S.D. | % (-) ROS ± S.D. | % (+) ROS ± S.D. | % (-) ROS ± S.D. | % (+) ROS ± S.D. | % (-) ROS ± S.D. |
| 0 hr | 4.8 ± 1.1 | 94.9 ± 1.1 | 15.8 ± 1.5 | 84.0 ± 1.4 | 9.8 ± 1.4 | 90.3 ± 1.6 |
| 24 hr | 4.4 ± 0.5 | 95.6 ± 0.5 | 7.7 ± 0.5 | 92.3 ± 0.5 | 9.2 ± 0.3 | 90.8 ± 0.3 |

Table S4 Total NO formation was measured using flow cytometer. Percentages of NO-positive KCs in cell population (live, dead, total) were represented as the mean ± S.D. of four independent experiments.

| % (+) NO ± S.D. in KCs | Non UVB-exposed KCs | | | 55 mJ/cm^2^ UVB -exposed KCs | | | Pretreated with the NAR +  55 mJ/cm^2^ UVB -exposed KCs | | |
| --- | --- | --- | --- | --- | --- | --- | --- | --- | --- |
|  | 0 hr | 10 hr | 24 hr | 0 hr | 10 hr | 24 hr | 0 hr | 10 hr | 24 hr |
| Live | 5.7 ± 2.0 | 7.2 ± 0.9 | 5.3 ± 1.2 | 4.7 ± 2.3 | 1.6 ± 0.2 | 1.4 ± 0.7 | 2.0 ± 0.5 | 1.5 ± 0.3 | 1.2 ± 0.8 |
| Dead | 2.1 ± 0.6 | 3.6 ± 1.1 | 8.7 ± 1.5 | 24.4 ± 2.0 | 28.4 ± 4.8 | 30.5 ± 5.3 | 18.6 ± 2.3 | 13.9 ± 4.8 | 13.6 ± 1.3 |
| Total | 7.8 ± 2.2 | 10.8 ± 1.7 | 14.0 ±1.5 | 29.1 ± 2.8 | 30.0 ± 4.6 | 31.9 ± 5.2 | 20.6 ± 2.1 | 15.4 ± 4.6 | 14.8 ± 1.2 |

Table S5 Percentages of cell cycle distribution were represented as the mean ± S.D. of three independent experiments. **P* < 0.05, ***P* < 0.01, compared with non UVB-exposed KCs, and *^##^P* < 0.01, compared with UVB-exposed KCs, using unpaired Student's t-test.

| Samples | % of cell cycle phases | | | | | |
| --- | --- | --- | --- | --- | --- | --- |
|  | At 10 hr | | | At 24 hr | | |
|  | G0/G1 (%) | S (%) | G2/M (%) | G0/G1 (%) | S (%) | G2/M (%) |
| Non UVB-exposed KCs | 62.6 ± 1.3 | 13.0 ± 1.2 | 23.9 ± 1.5 | 77.4 ± 1.3 | 7.9 ± 0.6 | 14.6 ± 1.0 |
| 55 mJ/cm^2^ UVB-exposed KCs | 59.6 ± 0.8* | 18.5 ± 0.4** | 21.5 ± 0.4 | 68.5 ± 0.9** | 10.9 ± 0.5** | 20.3 ± 0.7** |
| Pretreated with the NAR + 55 mJ/cm^2^  UVB-exposed KCs | 68.6 ± 0.9^##^ | 12.0 ± 0.8^##^ | 19.2 ± 1.6 | 76.1 ± 0.6^##^ | 7.8 ± 0.1^##^ | 16.0 ± 0.6^##^ |
